# APA-Scan: detection and visualization of 3′-UTR alternative polyadenylation with RNA-seq and 3′-end-seq data

**DOI:** 10.1186/s12859-022-04939-w

**Published:** 2022-09-28

**Authors:** Naima Ahmed Fahmi, Khandakar Tanvir Ahmed, Jae-Woong Chang, Heba Nassereddeen, Deliang Fan, Jeongsik Yong, Wei Zhang

**Affiliations:** 1 Department of Computer Science, University of Central Florida, 4000 Central Florida Blvd, Orlando, FL 32816 USA; 2 Department of Computer Engineering, University of Central Florida, 4000 Central Florida Blvd, Orlando, FL 32816 USA; 3 Department of Biochemistry, Molecular Biology and Biophysics, University of Minnesota Twin Cities, 420 Washington Ave. S.E., Minneapolis, MN 55455 USA; 4 School of Electrical, Computer and Energy Engineering, Arizona State University, 650 E Tyler Mall, Tempe, AZ 85287 USA

**Keywords:** Alternative polyadenylation, Transcriptome, RNA-seq, 3′-End-seq

## Abstract

**Background:**

The eukaryotic genome is capable of producing multiple isoforms from a gene by alternative polyadenylation (APA) during pre-mRNA processing. APA in the 3′-untranslated region (3′-UTR) of mRNA produces transcripts with shorter or longer 3′-UTR. Often, 3′-UTR serves as a binding platform for microRNAs and RNA-binding proteins, which affect the fate of the mRNA transcript. Thus, 3′-UTR APA is known to modulate translation and provides a mean to regulate gene expression at the post-transcriptional level. Current bioinformatics pipelines have limited capability in profiling 3′-UTR APA events due to incomplete annotations and a low-resolution analyzing power: widely available bioinformatics pipelines do not reference actionable polyadenylation (cleavage) sites but simulate 3′-UTR APA only using RNA-seq read coverage, causing false positive identifications. To overcome these limitations, we developed APA-Scan, a robust program that identifies 3′-UTR APA events and visualizes the RNA-seq short-read coverage with gene annotations.

**Methods:**

APA-Scan utilizes either predicted or experimentally validated actionable polyadenylation signals as a reference for polyadenylation sites and calculates the quantity of long and short 3′-UTR transcripts in the RNA-seq data. APA-Scan works in three major steps: (i) calculate the read coverage of the 3′-UTR regions of genes; (ii) identify the potential APA sites and evaluate the significance of the events among two biological conditions; (iii) graphical representation of user specific event with 3′-UTR annotation and read coverage on the 3′-UTR regions. APA-Scan is implemented in Python3. Source code and a comprehensive user’s manual are freely available at https://github.com/compbiolabucf/APA-Scan.

**Result:**

APA-Scan was applied to both simulated and real RNA-seq datasets and compared with two widely used baselines DaPars and APAtrap. In simulation APA-Scan significantly improved the accuracy of 3′-UTR APA identification compared to the other baselines. The performance of APA-Scan was also validated by 3′-end-seq data and qPCR on mouse embryonic fibroblast cells. The experiments confirm that APA-Scan can detect unannotated 3′-UTR APA events and improve genome annotation.

**Conclusion:**

APA-Scan is a comprehensive computational pipeline to detect transcriptome-wide 3′-UTR APA events. The pipeline integrates both RNA-seq and 3′-end-seq data information and can efficiently identify the significant events with a high-resolution short reads coverage plots.

**Supplementary Information:**

The online version contains supplementary material available at 10.1186/s12859-022-04939-w.

## Introduction

Poly(A)-tails are added to pre-mRNA after the polyadenylation signal (PAS) during the 3′-end processing of pre-mRNA [1]. The last exon of mRNA contains a non-coding region, 3′-untranslated region (3′-UTR), which spans from the termination codon to the polyadenylation site. The 3′-UTR acts as a molecular scaffold to bind microRNAs and RNA-binding proteins and functions in regulatory gene expression [2]. In human and mouse, more than 70% of genes contain multiple PASs in their 3′-UTRs and polyadenylation using upstream PASs leads to the production of mRNA with shortened 3′-UTRs (3′-UTR APA) [3, 4]. 3′-UTR APA is known to increase the efficiency of translation and is associated with T cell activation, oncogene activation, and poor prognosis in many diseases [5–7]. Recent study has demonstrated that 3′-UTR APA is one way to increase protein synthesis without increasing the quantities of mRNAs, indicating that it is an important element in gene expression which cannot be understood by conventional differential gene or transcript expression analysis [8]. Up-regulation of mTOR signaling pathway can lead to transcriptome-wide 3′-UTR APA [8, 9].

3′-UTR APA has gained much attention recently and the importance of the 3′-UTR APA in human diseases has been demonstrated as mentioned above. Some recent studies show that both proliferating cells and transformed cells favor expression of shorter 3′-UTR through APA and lead to the activation of oncogenes [6, 10]. Some other research shows the trend in cancer cells for highly expressed genes to exhibit shorter 3′-UTR with fewer microRNA binding sites, decreasing microRNA-mediated translation repression [5, 11]. All these studies imply that 3′-UTR APA may serve as a new layer of prognostic biomarker. A scalable computational model is highly needed to detect the genome-wide unannotated 3′-UTR APA in different phenotypes.

**Fig. 1:**
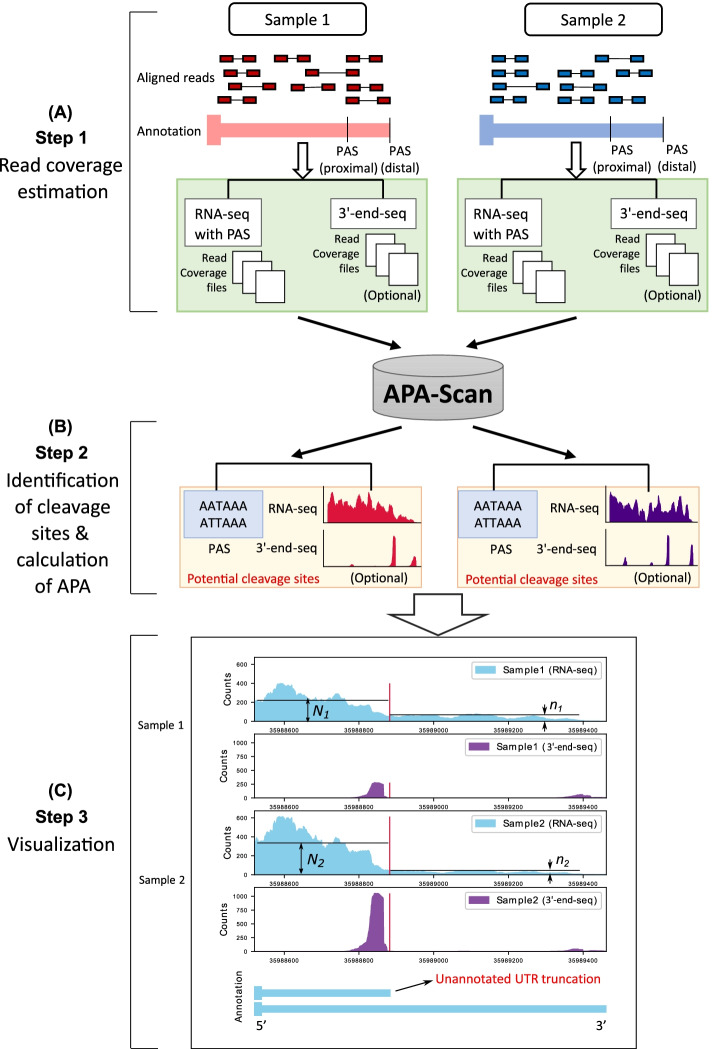
Workflow of APA-Scan. Starting with aligned RNA-seq and 3′-end-seq (optional) bam files, APA-Scan consists of three steps and generates high quality graphical illustration of aligned sequences with the indication of 3′-UTR APA events. **A** Read coverage files are generated for RNA-seq and 3′-end-seq (if provided) input samples. **B** APA-Scan identifies potential cleavage sites according to polyadenylation signal (PAS) hexamer: ATTAAA or AATAAA, or 3′-end peaks (if 3′-end-seq data is available). **C** Graphical illustration of the identified events. The illustration also highlights unannotated short 3′-UTR transcript identified from this task. The vertical red lines show the corresponding cleavage sites

Several bioinformatics pipelines are available for the analysis of UTR-APA using RNA-seq data [12–16]. In general, all these methods measure the changes in 3′-UTR lengths by modeling the RNA-seq read density change near the 3′-end of mRNAs. Indeed, with the aid of these methods, RNA-seq experiments became a powerful approach to investigate 3′-UTR APA. However, in many cases the identified APA sites are not functionally and physiologically relevant because most pipelines do not reference actionable PASs in their 3′-UTR APA simulation. RNA-seq is not particularly accurate when it comes to identifying polyadenylation sites, making novel APA transcript identification rather difficult. Therefore, 3′-end-seq data has been developed to address these issues by enriching for 3′-end reads in high-throughput sequencing experiment [17] and provides the accurate polyadenylation sites. In addition to the limitations of the current bioinformatics pipelines mentioned above, none of them can provide high-resolution read coverage plots of the APA events with an accurate annotation. We have developed APA-Scan (Fig. 1), a bioinformatics program, to detect and visualize genome-wide 3′-UTR APA events. APA-Scan integrates both 3′-end-seq data and the location information of predicted canonical PASs with RNA-seq data to improve the quantitative definition of genome-wide UTR APA events. APA-Scan efficiently manages large-scale alignment files and generates a comprehensive analysis for UTR APA events. It is also advantageous in producing high quality plots of the events.

## Results

APA-Scan is designed to identify both annotated and de novo 3′-UTR APA events between different biological conditions. To access the performance of APA-Scan, it was compared with two baseline methods on both simulated and real RNA-seq datasets. In the simulation experiment, we first generated synthetic dataset with pre-defined 3′-UTR APA events (ground truth) to test if the APA-Scan and baseline methods can detect them. Next, we performed experiments on two mouse embryonic fibroblast (MEF) cells to evaluate the performance of APA-Scan. The results of analyzing real MEF RNA-seq datasets were validated using both qPCR and 3′-end-seq data.

### Experimental results with simulated RNA-seq data

In the simulation experiment, we generated synthetic RNA-seq short reads with flux-simulator [18]. 1000 pre-defined 3′-UTR APA events were simulated as the ground truth between two different conditions. In each condition, three technical replicates were generated by repeating the experiment three times with the same parameter setting in the flux simulator. The details of the parameters used in this experiment are provided in the Additional file 2. For both conditions, the gene expressions were sampled from a Poisson distribution to reflect a real RNA-seq data [19]. For each gene, one proximal polyadenylation site was synthesized to represent the end of the short isoform and the end of the annotated transcript was applied to define the end of the long isoform of that gene. To generate the ground truth profile of the 3′-UTR APA events, the expression proportions of the short and long isoforms in the same gene were assigned significantly different values in two conditions (i.e., the proportion difference was larger than 10%) to represent the existence of the APA event (Table 1).

**Table 1:** Categorized overview of the technical parameters of APA-Scan

Categories	Types	Description
Input data	APA-Scan^*peaks*^	Both 3′-end-seq and RNA-seq data are provided
	APA-Scan^*PAS*^	Only RNA-seq data is provided
Mode	Default	Search for shorter 3′-UTR APA events
	Extended	Search for longer 3′-UTR APA events
Reported list	Default	List the most significant cleavage site for each gene
	All	List all candidate cleavage sites for each gene

**Fig. 2:**
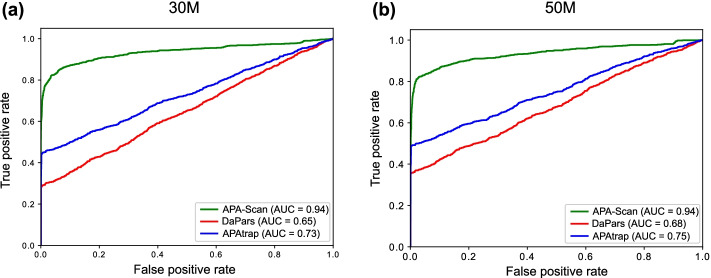
Simulation experiment to assess the performance of APA-Scan and the baseline methods (DaPars and APAtrap). **a** Results on the simulation experiment with 30 million (30 M) short reads. **b** Results on the simulation experiment with 50 M short reads. The receiver operating characteristic (ROC) curves, i.e., true positive rate against false positive rate, are plotted

In the simulation experiment, two sets of synthetic data were generated by flux-simulator. One with 30 M (30 million) paired-end reads in each replicate and one with 50 M paired-end read. In both cases, the read length is 76 bps of each end. APA-Scan was compared with DaPars and APAtrap on the simulated RNA-seq datasets. To detect the significant 3′-UTR APA events, APA-Scan used p-value < 0.05 (χ^2^-test) as the cutoff. DaPars identified APA events according to the difference in PDUI (Percentage of Distal polyA Usage Index) values between two conditions > 0.1 and FDR < 0.05; whereas APAtrap selected events using the cutoff values of two parameters: percentage difference of APA site usage between two conditions > 0.1 and FDR < 0.05. The performance of the methods is then evaluated using AUC score, sensitivity and specificity. Figure 2 shows that, APA-Scan outperformed the two baselines in terms of AUC scores and got the best score of 0.94 in both sequence depths (30 M reads and 50 M reads) and followed by APAtrap (0.73 in 30 million reads case and 0.75 in 50 million reads case). DaPars did not work very well compared to the other two methods and the AUC scores were below 0.7 in both cases, though there was an improvement in the case with more reads. We also report the sensitivity and specificity for each method with two different sequencing depths in Table 2. APA-Scan gets the highest sensitivity and specificity scores for both cases, which indicates that APA-Scan outperformed the baseline methods in detecting the true 3′-UTR APA events and eliminating the true negative ones.

**Table 2:** Comparison among APA-Scan, DaPars and APAtrap on simulated RNA-seq data with two different sequencing depths (30 million reads and 50 million reads)

Reads	Method	AUC	Sensitivity	Specificity
30 M	APA-Scan	**0.94**	**0.83**	**0.95**
	DaPars	0.65	0.33	0.58
	APAtrap	0.73	0.57	0.90
50 M	APA-Scan	**0.94**	**0.83**	**0.95**
	DaPars	0.68	0.41	0.71
	APAtrap	0.75	0.63	0.91

AUC (the area under the ROC curve) score, sensitivity, specificity of the three methods are reported. The best results across the three methods are bold

**Fig. 3:**
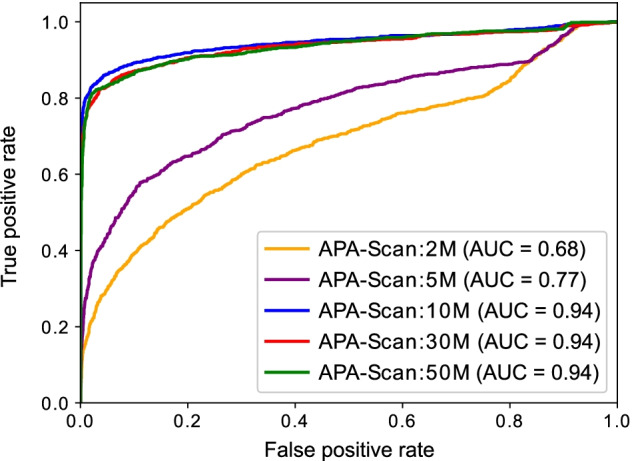
Simulation experiment to assess the performance of APA-Scan on different sequencing depths. The ROC curves for the results of different RNA-seq read depth are plotted

As different sequencing depths may affect the performance of APA-Scan, we generated five simulation experiments with different read depths, i.e., 2 M, 5 M, 10 M, 30 M, and 50 M paired-end reads by flux-simulator with the same parameter setting to learn the impact of sequencing depths in the analysis of 3′-UTR APA with APA-Scan. In this experiment, the read length was also 76 bps for each end and three replicates were generated for each condition in each read depth using the same procedures as mentioned in the previous section. Figure 3 shows the ROC curves for different sequencing depth on detecting the 3′-UTR APA events. APA-Scan shows moderate performance with low sequencing depths (i.e., 2 M and 5 M). However, the performance of APA-Scan improved drastically (AUC = 0.94) after it reached to a certain sequencing depth (i.e., 10 M in this study) and holds that performance across read depths above that threshold. This result suggests that APA-Scan is quite robust in detecting APA events on lowly expressed genes and relatively low read coverage samples.

### Experimental results with MEFs samples

**Fig. 4:**
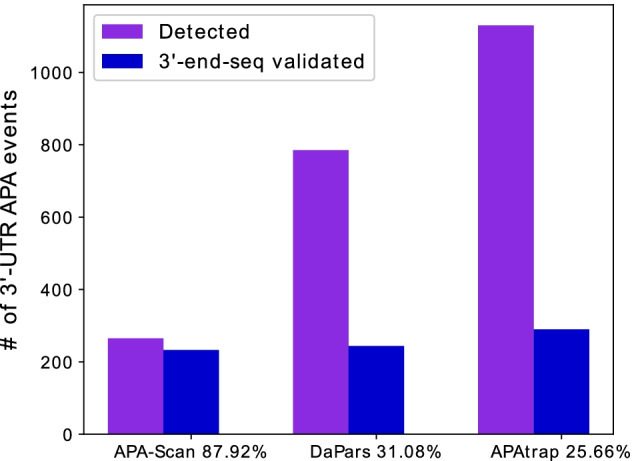
Evidence of polyadenylation sites supported by 3′-end-seq data for 3′-UTR APA events detected by different methods in MEFs samples. The number of events predicted by each method are shown in purple and the number of events validated by the signals in the 3′-end-seq data are shown in blue. The x-axis shows the percentage of the identified events is validated by 3′-end-seq

**Fig. 5:**
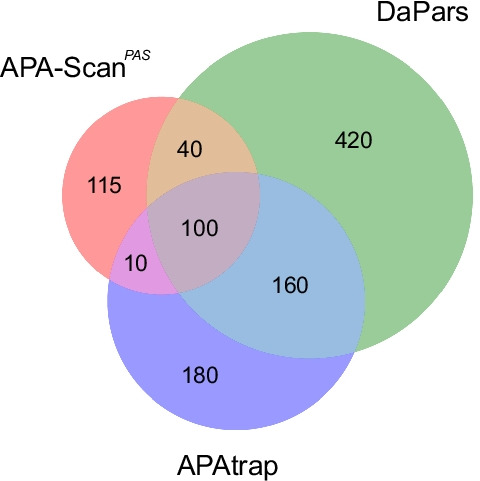
Venn diagram shows the overlapped genes with the 3′-UTR APA events identified by three methods (i.e., APA-Scan, DaPars and APAtrap) between two MEFs samples (WT vs Tsc1^−/−^)

In the real RNA-seq experiments, two MEFs samples Tsc1^−/−^ and WT were used in the analysis to evaluate the performance of APA-Scan and baseline methods. Knockout of Tsc1, a negative regulator of mTOR pathway, leads to uncontrolled mTOR hyper-activation compared with WT. For the comparison and evaluation purposes, the APA-Scan was run on two different setups. One used PASs in the 3′-UTRs as potential cleavage sites, and we denote it by APA-Scan^*PAS*^. The other one considered 3′-end-seq peaks as candidate sites, and it is denoted as APA-Scan^*peaks*^. First, APA-Scan^*PAS*^ was applied to detect 3′-UTR APA events between the two MEFs samples with p-value < 0.05. APA-Scan^*PAS*^ detected 265 events, whereas DaPars and APAtrap detected 785 and 1130 significant events, respectively. These events were then verified by the polyadenylation sites reported by 3′-end-seq data. If a predicted 3′-UTR APA event is within 50 bps upstream or downstream of the loci of the peak(s) in 3′-end-seq data, then this APA event is considered overlapping with the 3′-end-seq signals. Though APA-Scan^*PAS*^ detected less number of significant events compared to the baseline methods, 87.92% (233) of the events were validated by the 3′-end-seq signals according to the result shown in Fig. 4 and Table 3. DaPars and APAtrap identified more events than APA-Scan^*PAS*^, however, both the number and ratio of the overlapping events with the 3′-end-seq signals are significantly lower than the events detected by APA-Scan^*PAS*^. Note that APA-Scan^*PAS*^ did not use any information from 3′-end-seq data to identify the APA events. These results concur with our findings in the simulation experiment that APA-Scan not only do better detection on the true APA events but also prevent the false positives. Figure 5 shows the number of overlapped genes with the 3′-UTR APA events detected by the three methods. From the results, we can conclude that the agreement of the three methods is not high and most identified events were only detected by one method.

**Table 3:** Number of events detected by APA-Scan, DaPars and APAtrap and validated by 3*′*-end-seq data for MEFs samples

Method	Detected	Validated by 3′-end-seq	Ratio (%)
APA-Scan	265	233	87.92
DaPars	785	244	31.08
APAtrap	1130	290	25.66

**Fig. 6:**
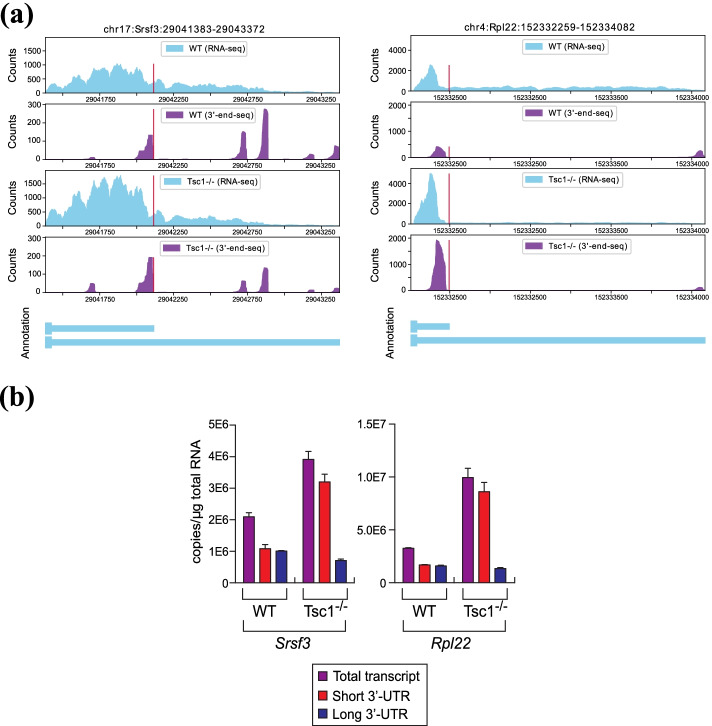
Experimental results: **a** RNA-seq and 3′-end-seq read coverage plots of the 3′-UTR in *Srsf3* and *Rpl22* gene in the two samples with isoform annotation. **b** The level of total, short 3′-UTR, and long 3′-UTR transcripts from *Srsf3* and *Rpl22* was measured by qPCR. Because it is not possible to design specific primers for the qPCR analysis of short 3′-UTR transcript, the amount of short 3′-UTR transcripts were calculated by subtracting the quantity of long 3′-UTR transcripts from total

To further validate the analysis results by APA-Scan, we conducted qPCR experiments for Srsf3 and Rpl22 transcripts from Tsc1^−/−^ and WT MEFs based on the significant 3′-UTR APA events reported by APA-Scan^*peaks*^. These genes were selected due to the design of PCR (polymerase chain reaction) primers for wet-lab validation. As shown in Fig. 6, both Srsf3 and Rpl22 showed the increase of the short 3′-UTR transcript by APA in Tsc1^−/−^ compared to WT MEFs, which is consistent with our observations on the RNA-seq and 3′-end-seq read coverage plots. These results further confirm that APA-Scan can identify the true 3′-UTR APA events with RNA-seq and 3′-end-seq samples from two different biological contexts. The more details of the qPCR analysis and the primer sequences of the two genes are available in the Additional file 1.

**Fig. 7:**
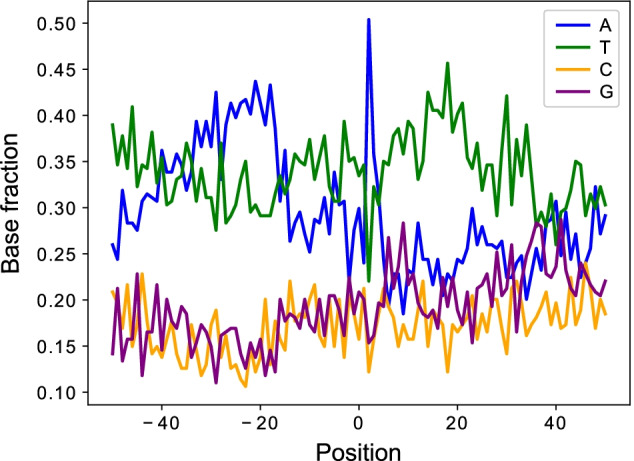
Nucleotide composition of the sequence surrounding the polyadenylation sites identified by APA-Scan^*peaks*^ for MEFs samples. 50 bp up and downstream region is plotted with base sequences. x-axis denotes the position in the region, 0 is the location of the identified polyadenylation site. y-axis shows the fraction of the nucleotides content at each position

Generally, the nucleotide profiles surrounding the polyadenylation sites are dominated by two motifs and their variants: AATAAA and ATTAAA and these two hexamers are observed upstream of the cleavage sites [20]. This phenomenon leads us to explore the nucleotide composition near the predicted polyadenylation sites by APA-Scan^*peaks*^. Figure 7 shows a high concentration/cluster of nucleotide ‘A’ in the polyadenylation site, positioned at 0. The upstream surrounding region is also dominated by ‘A’ and ‘T’, which clearly indicates the existence of potential 3′-UTR APA events.

## Discussion

APA is one mechanism for post-transcriptional regulation of mRNA expression, and it is defined as use of more than one polyadenylation sites. 3′-UTR APA is one of the most frequent APA forms, which contains more than one polyadenylation sites in the 3′-UTR. It generates multiple mRNA transcripts with different 3′-UTR lengths without affecting the protein encoded by the gene. Since the 3′-UTR of mRNA of-ten contains binding sites for microRNAs, 3′-UTR APA potentially leads to altered mRNA stability or protein translation efficiency due to variation of 3′-UTR length. Identification and assessment of APA sites has been a major goal in understanding transcriptomic diversity. Several bioinformatics tools have been developed to predict transcriptome-wide polyadenylation sites with RNA-seq data. However, our experimental results on simulated and real samples indicate that the current methods (e.g., DaPars and APAtrap) can detect large number of APA events, but significant portion of the events are false positives. A similar data analysis on BT549 breast cancer cells (mock vs. torin 1 treated) in Additional file 1: Fig. S1 illustrates a similar pattern. By integrating 3′-end-seq and RNA-seq data, APA-Scan can potentially reduce the number of false positive events. To evaluate the performance of APA-Scan on real cancer patient samples, one pair (tumor vs. matched normal tissue) of The Cancer Genome Atlas (TCGA) breast cancer samples are also analyzed and reported in the Additional file 1. Additional file 1: Fig. S2 shows that a significant portion (> 72%) of the 3′-UTR APA events are not differentially expressed. Therefore, APA-based molecular signatures could provide additional predictive power of cancer outcomes by combining the differently expressed genes.

APA-Scan not only can accurately detect the splicing events compared to the baseline methods, but also provides reasonable running time. Table 4 shows a comparison of the CPU time of each method on Tsc1^−/−^ and WT MEFs. The CPU time was measured on an Intel(R) Xeon(R) CPU E5-2620 v4 @ 2.10 GHz machine. Both APA-Scan and APAtrap completed the analysis in a similar amount of time. However, DaPars is much slower than the other two methods which is not suitable to be applied on large-scale experiment in terms of running time. Overall, this study reports an efficient and precise framework for 3′-UTR APA identification with RNA-seq and 3′-end-seq data.

**Table 4:** CPU time of APA-Scan, DaPars and APAtrap on two MEFs samples (WT vs Tsc1^−/−^)

APA-Scan	DaPars	APAtrap
48 min	21 h	36 min

## Conclusion

We developed APA-Scan, which offers a comprehensive computational pipeline to identify transcriptome-wide 3′-UTR APA events. By integrating RNA-seq data and 3′-end-seq information (experimentally verified or computationally predicted). APA-Scan can efficiently identify significant APA events and also, can illustrate the events with read coverage plots. 3′-end-seq signals and the wet-lab experiment using qPCR demonstrate that APA-Scan provides high-accuracy and quantitative profiling of 3′-UTR APA events. Therefore, we expect that, APA-Scan will serve as a useful tool for APA site analysis.

## Methods

### APA-Scan pipeline

APA-Scan workflow comprises of three major steps: (i) read coverage estimation; (ii) identification of polyadenylation sites and the calculation of APA; (iii) graphical illustration of UTR APA events (Fig. 1). First, APA-Scan takes aligned RNA-seq and 3′-end-seq data from two different biological conditions as input. Each biological condition can have multiples samples or replicates. The read coverage files are generated by SAMtools [21]. In this step, the 3′-end-seq data is an optional input.

In the second step, APA-Scan starts the analysis by extracting 3′-UTR frames for each gene. APA-Scan is designed in two modes: (a) Default, and (b) Extended. All the aligned reads from 3′-end-seq data are pooled together to identify peaks and the corresponding unannotated cleavage sites in 3′-UTR regions and downstream of the 3′-UTR regions (i.e., APA-Scan^*peaks*^). In the Default mode, 3′-UTR regions are selected according to the end of the longest annotated transcript of the gene. The loci of peaks identified in the 3′-end-seq data are considered as potential cleavage sites. If the 3′-end-seq data is not provided by the user, detected PASs (generally two variations of the hexamers: AATAAA, ATTAAA) in 3′-UTRs are considered as the potential cleavage sites (i.e., APA-Scan^*PAS*^) follow the ideas in Omni-PolyA [16] which use 12 most common PAS variants to determine the cleavage sites. In the Extended mode of APA-Scan, the potential peaks/PAS signals are searched up to 10 kb downstream of the end of transcript to discover de novo distal polyadenylation sites. The locations detected from all input samples are merged to get a combined list of potential cleavage sites. The major commands and general terminologies to run APA-Scan are listed in Table 1.

APA-Scan evaluates each empirical cleavage site in the 3′-UTR of a transcript by contrasting the RNA-seq short reads coverage up and downstream of the candidate site between the two biological conditions. n and N denote the average read coverage up and downstream of the site. They are determined by estimating the number of reads mapped to upstream and downstream of the cleavage site, *r*_*u*_ and *r*_*d*_, divided by their effective length, *l*_*u*_ and *l*_*d*_, respectively (i.e., $n = \frac{{r_{u} }}{{l_{u} }}$ and $N = \frac{{r_{d} }}{{l_{d} }}$). For each potential polyadenylation site, the ratio differences between the samples in two conditions are calculated based on the following equation.

$\frac{{n_{1} }}{{N_{1} }} - \frac{{n_{2} }}{{N_{2} }},$ where 1 and 2 represent the two conditions. Ratio difference indicates the change in read coverage between two conditions and only the absolute ratio difference > 0.1 is considered as candidate site for the further analysis. After that, the canonical 2 × 2 χ^2^-test is applied to report the p-value for each candidate site. The χ^2^-test measures how much the observation deviates from the null hypothesis. In our experiment, we set the null hypothesis as the average read coverage before and after the cleavage sites are consistent among the two biological conditions. For any true 3′-UTR APA event, there must be a significant read coverage drop-off around the cleavage sites, and the ratios of the average read coverages before and after the cleavage sites are crucially different in the two conditions. In such cases, the χ^2^-test precisely reports significant p-values to reject our null hypothesis. APA-Scan will report both significant and insignificant in an Excel file. A comprehensive user’s manual is provided in the Additional file 2.

In the third step, based on the significance of 3′-UTR APA events calculated in the previous step, APA-Scan generates RNA-seq and 3′-end-seq (if provided) read coverage plots with the 3′-UTR annotations for one or more user-specific events. Users may specify the region of the genome locus to generate the read alignment plot. Figure 1 (Step 3) illustrates an example of the read coverage plot generated by APA-Scan.

### Baselines and evaluation methods

In this study, two widely used 3′-UTR APA identification approaches, DaPars [12] and APAtrap [15] were applied to compare the performance with APA-Scan. The command lines to run the baseline methods are available in the Additional file 1. To evaluate the performance of APA-Scan and baseline methods, the area under the ROC curve (AUC), sensitivity and specificity were used on the identified lists of 3′-UTR APA events.

### Short read alignments and peak identification

In this study, two mouse embryonic fibroblasts (MEFs) samples and two breast cancer cell lines (BT549) were used in the analysis to evaluate the performance of APA-Scan and baseline methods. For the MEFs samples, we performed RNA-seq and 3′-end-seq analyses of poly(A +) RNAs isolated from Tsc1^−/−^ and wild-type (WT) MEFs. In the RNA-seq analysis, 63,742,790 paired-end reads for WT and 74,251,891 paired-end reads for Tsc1^−/−^ MEFs were produced from Hi-Seq pipeline with length of 50 bps of each end. The short reads were aligned to the mm10 reference genome by TopHat2 [22], allowing up to two mismatches. Finally, 87.1% of short reads from WT and 87.5% of sequence reads from Tsc1^−/−^ MEFs were mapped to the reference genome for APA analysis in the study. In the 3′-end-seq analysis, the reads from WT and Tsc1^−/−^ MEFs were preprocessed to trim A’s off the 3′-ends and then filtered by removing the reads of low-quality 3′-end (Phred score < 30) and shorter than 25 bps. The remaining reads were aligned to the mm10 reference genome by Bowtie [23] without allowing any mismatches. In total, 6,186,893 paired-end reads were aligned for WT and 5,382,111 reads were aligned for Tsc1^−/−^. All aligned reads from 3′-end-seq were pooled together in order to identify peaks and the corresponding cleavage sites in the reference genome by the read coverage signals. In each read alignment ‘hill’, the location with the highest read coverage between two zero coverage positions was considered as the peak of the ‘hill’. The 3′-end of the peak is chosen as the potential corresponding cleavage sites where the read coverage at the peak quantifies the cleavage at the site. For the breast cancer cell lines, we performed RNA-seq analysis of poly(A +) RNAs isolated from BT549 mock and Torin1 treated cells. 131,955,082 paired-end reads for BT549 mock, and 138,127,113 paired-end reads for BT549 treated with Torin1 were produced from Hi-Seq pipeline with length of 51 bps of each end. The short reads were aligned to the hg38 reference genome by TopHat2, allowing up to two mismatches. Finally, 85.2% of short reads from BT549 mock and 84.7% of sequence reads from BT549 treated with Torin1 were mapped to the reference genome for APA analysis in the study.

## Supplementary Information

**Additional file 1. Fig. S1 and Fig. S2**: The command lines used for running the baseline methods; Parameters to run flux-simulator; qPCR analysis and primer sequences.

**Additional file 2.** User’s manual of APA-Scan.

## Data Availability

The source code in this study is available at: https://github.com/compbiolabucf/APA-Scan. The accession number for the MEFs RNA-seq data in this study is SRP056624. The accession number for the 3′-end-seq data in this study is SRP133833.

## Abbreviations

APA: Alternative polyadenylation
3′-UTR: 3′-Untranslated region
mTOR: Mechanistic target of rapamycin
PAS: Polyadenylation signal
MEFs: Mouse embryonic fibroblasts
WT: Wild-type
ROC: Receiver operating characteristic
AUC: Area under the ROC curve
qPCR: Quantitative polymerase chain reaction
