# Supplementary material for: APA-Scan: detection and visualization of 3′-UTR alternative polyadenylation with RNA-seq and 3′-end-seq data
Source: BMC Bioinformatics. 2022 Sep 28;23(Suppl 3):396. doi: 10.1186/s12859-022-04939-w (PMC9520800; doi:10.1186/s12859-022-04939-w)
Supplement: Supplementary file 1 — Additional file 1. Fig. S1 and Fig. S2: The command lines used for running the baseline methods; Parameters to run flux-simulator; qPCR analysis and primer sequences. [file 12859_2022_4939_MOESM1_ESM.pdf]

APA-Scan: Detection and Visualization of 3'-UTR APA with RNA-seq and 3'-end-seq Data

Naima Ahmed Fahmi, Khandakar Tanvir Ahmed, Jae-Woong Chang, Heba Nassereddeen, Deliang Fan, Jeongsik Yong and Wei Zhang

June 29, 2021

1 Running baselines

1.1 DaPars

Inputs:

- BED files. Flux-simulator simulated three fastq files for three replicates in each condition. Using SAMtools (v0.1.8), read coverage files for each chromosome are generated in BAM format from the fastq files. Six bedgraph files in two conditions are generated from the BAM files using BEDtools.
- Gene annotation in .bed format: mm10_Refseq.bed
- a configuration file: configure.txt

```
Annotated_3UTR=mm10_Refseq_extracted_3UTR.bed
Group1_Tophat_aligned_Wig = case_1.bedgraph, case_2.bedgraph, case_3.bedgraph
Group2_Tophat_aligned_Wig = control_1.bedgraph, control_2.bedgraph, control_3.bedgraph
Output_directory = DaPars_out/
Output_result_file = Dapars_out
Num_least_in_group1 = 1
Num_least_in_group2 = 1
Coverage_cutoff = 30
FDR_cutoff = 0.05
PDUI_cutoff = 0.1
Fold_change_cutoff = 0.59
```

Command 1:
python DaPars_Extract_Anno.py -b mm10_Refseq.bed
-s mm10_Refseq_id.txt -o mm10_Refseq_extracted_3UTR.bed
Command 2:
python DaPars_main.py configure.txt

1.2 APAtrap

Inputs:

- BED files. Flux-simulator simulated three fastq files for three replicates in each condition. Using SAMtools (v0.1.8), read coverage files for each chromosome are generated in BAM format from the fastq files. Six bedgraph files in two conditions are generated from the BAM files using BEDtools.
- Gene annotation in .bed format: mm10_Refseq.bed

Command 1:
identifyDistal3UTR -i A1.bedgraph A2.bedgraph A3.bedgraph B1.bedgraph B2.bedgraph B3.bedgraph -m mm10_Refseq.bed -o mm10.utr.bed
Command 2:
predictAPA -i A1.bedgraph, A2.bedgraph, A3.bedgraph B1.bedgraph, B2.bedgraph, B3.bedgraph -g 2 -n 3 3 -u mm10.utr.bed -o APA_output.txt
Command 3:
deAPA('APA_output.txt', 'APA_output.stat.txt', 1, 2, 1, 1, 20)

2 Parameters to run flux-simulator (30 million reads)

Parameters	Value	Description
REF_FILE_NAME	mm10.refGene.gtf	GTF reference annotation
GEN_DIR	Genome_mm10	Genomic sequences directory
NB_MOLECULES	8000000	Number of RNA molecules
TSS_MEAN<br>POLYA_SCALE<br>POLYA_SHAPE	100<br>100<br>2	Transcript modification parameters
FRAG_SUBSTRATE<br>FRAG_METHOD<br>FRAG_NB_LAMBDA<br>FRAG_NB_M	DNA<br>NB<br>575<br>1	Library Preparation parameters
RTRANSCRIPTION	YES	Switch on reverse transcription
PCR_DISTRIBUTION<br>GC_MEAN<br>GC_SD<br>PCR_PROBABILITY	none<br>NaN<br>NaN<br>0.1	Amplification parameters
FILTERING	YES	Switches size selection On
UNIQUE_IDS	TRUE	Create Unique Read Identifiers for paired-end
READ_NUMBER	30000000	Number of reads
READ_LENGTH	76	Length of each read
PAIRED_END	YES	Paired end reads
FASTA	YES	Generate Fasta file
ERR_FILE	76	Error model for length 76

3 Realtime quantitative PCR (RT-qPCR) analysis and primer sequences

Realtime quantitative PCR (RT-qPCR) analysis and primer sequences: Total RNAs from TSC1 WT or TSC1-/- MEF cells were isolated by Trizol method according to manufacturer's protocol (https://assets.thermofisher.com/TFS-Assets/LSG/manuals/trizol_reagent.pdf). Reverse transcription reaction using Oligo-d(T) priming and NxGen M-MuLV Reverse transcriptase (Lucigen) was carried out according to the manufacturer's protocol (https://www.lucigen.com/docs/manuals/MA115-M-MuLV.pdf). SYBR Green was used to detect and quantitate the PCR products in real-time reactions. Quantitation of the real-time PCR results was done using standard curve method for accuracy and reliability of the analysis. The primer sequences used to measure the RSI for each transcript are as follows:
mRpl22 Total forward 5'-AAGTTCAC CCTGGACTGC AC-3'
mRpl22 Total reverse 5'-GTGATCTT GCTCTTGCTG CG-3'
mRPL22 Long Forward 5'-TGGGCATC TGGGCTTTTA GG-3'
mRPL22 Long reverse 5'-GCTTGTTGCA GACTTGCTCA-3'
mSRSF3 Total forward 5'- GCTGCCGTGTAAGAGTGGAA-3'
mSRSF3 Total reverse 5'- AGGACTCCTCCTGCGGTAAT-3'
mSRSF3 Long forward 5'- TGCAACAGTCTTGTGGCTTA-3'
mSRSF3 Long reverse 5'-TGCAATGGCTCTTACATAGACC-3'

4 Venn diagram for BT549 mock vs BT549 Torin1 treated

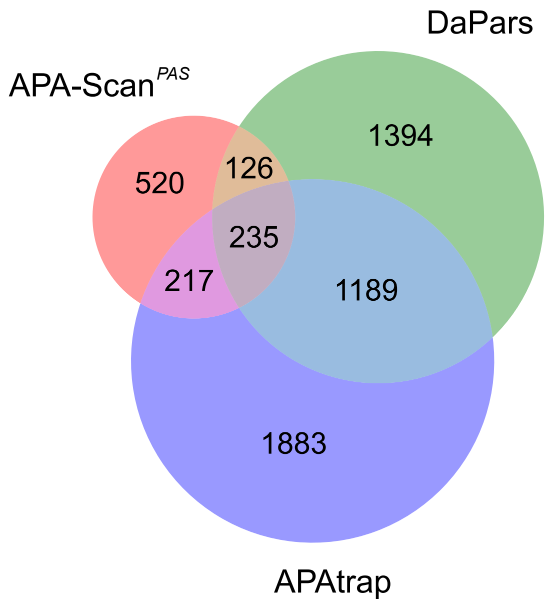

Figure S1: Venn diagram shows the overlapped genes with the 3'-UTR APA events identified by three methods between two breast cancer cell lines (BT549 mock vs BT549 Torin1 treated).

5 Experimental results with TCGA BRCA samples

One pair of TCGA breast cancer normal and tumor samples were selected and ran through APA-Scan for the detection of 3'-UTR APA. A total of 1266 APA events were detected between the two samples, whereas 1170 (92%) significant APA events were identified in tumor and 96 (8%) significant APA events were found in normal tissue sample. To inspect the correlation between 3'-UTR APA events and the gene expression profiles, we did the differential gene expression analysis and identified the genes in both tumor and normal samples. The result is illustrated in Figure S2. In the scatter plot, the y-axis denotes the Log_2 fold-change in the differential gene expression analysis and the x-axis shows the significance of UTR-APA (Log_{10} p-value). The left three sections and the right three sections show the 3'-UTR truncated genes in tumor and normal sample, respectively. The top three sections and the bottom three sections represent the up-regulated and down-regulated genes in normal tissue over tumor sample. This plot leads us to the observation that majority (>72%) of the 3'-UTR APA genes are not differentially expressed.

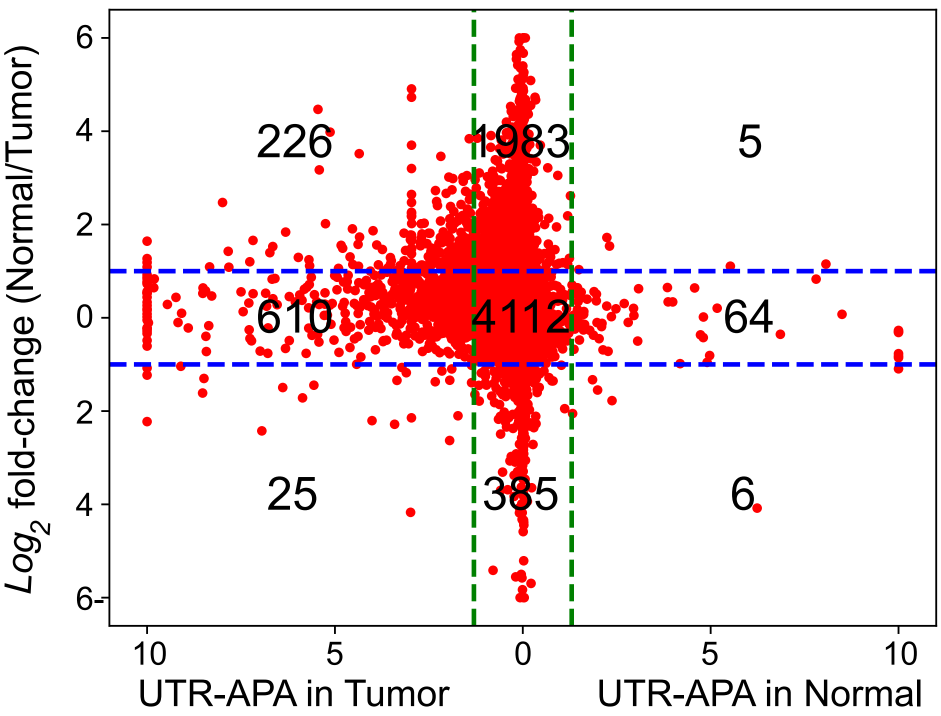

Figure S2: Scatter plot of APA and differentially expressed genes for TCGA tumor (TCGA-BH-A0BQ-01A) vs. matched normal tissue (TCGA-BH-A0BQ-11A) samples. Red dots represent individual gene in the analysis. Horizontal blue-dashed lines represent the cutoff values for two-fold changes in differential gene expression. Vertical green-dashed lines represent the cutoff values for $log_{10}(p\text{-value})$ of 3'-UTR APA determined by the Chi-squared test.
