# Supplementary material for: APA-Scan: detection and visualization of 3′-UTR alternative polyadenylation with RNA-seq and 3′-end-seq data
Source: BMC Bioinformatics. 2022 Sep 28;23(Suppl 3):396. doi: 10.1186/s12859-022-04939-w (PMC9520800; doi:10.1186/s12859-022-04939-w)
Supplement: Supplementary file 2 — Additional file 2. User’s manual of APA-Scan. [file 12859_2022_4939_MOESM2_ESM.pdf]

APA-Scan User Manual

1 About

APA-Scan is a computational tool which can detect and visualize genome-wide 3'-UTR APA events. APA-Scan integrates both 3'-end-seq (an RNA-seq method with a specific enrichment of 3'-ends ofmRNA) data and the location information of predicted canonical PASs with RNA-seq data to improve the quantitative definition of genome-wide UTR-APA events. It is also advantageous in producing high quality plots of the user defined events.

2 Download

APA-Scan is downloadable directly from https://github.com/compbiolabucf/APA-Scan. Users need to have python (version 3.0 or higher) installed in their machine to run APA-Scan.

3 Required Softwares

1. Python (version 3.0 or higher)
2. Samtools 0.1.8* [This specific version]

Required python packages

1. Pandas: $ pip install pandas
2. Bio: $ pip install biopython
3. Scipy: $ pip install scipy
4. Numpy: $ pip install numpy
5. Peakutils: $ pip install PeakUtils

4 Run APA-Scan

APA-Scan can handle both human and mouse data for detecting potential APA truncation sites. The tool is designed to follow the format of Refseq annotation and genome file from UCSC Genome Browser. Users need to have the following two files in the parent directory in order to run APA-Scan:

1. Refseq annotation (.txt format)
2. Genome fasta file (downloaded from UCSC genome browser)

4.1 Required files

APA-Scan has two python scripts: **APA-Scan.py, Make-Plots.py**
And 1 configuration file: **configuration.ini**

The configuration file allows the user to specify the directories of the input samples, the species to be analyzed and the directory where all output files will be stored.
APA-Scan supports the analysis of multiple samples that belong to two different groups- all BAM files inside the input1 directory will be considered as part of the first group, and all BAM files inside

the input2 directory will be considered as part of the second group. It is required to have at least one BAM file in each input directory.

4.2 Running with parameters in the configuration.ini file

(refers to a mandatory field)*

species*:	Species name (human/mouse)
input1* :	Directory containing the first group of samples with RNA-seq data [must be a folder name without '/' at the end]
input2* :	Directory containing the second group of samples with RNA-seq data[must be a folder name without '/' at the end]
pas1* :	Directory containing the first group of samples with 3'-end-seq data [must be a folder name without '/' at the end]. Default is NULL
pas2* :	Directory containing the second group of samples with 3'-end-seq data [must be a folder name without '/' at the end]. Default is NULL
extended* :	APA-Scan will run on 'Extended 3UTR' mode and it will search for APA sites upto 10kb downstream of the annotated transcript. Value: yes or no
All* :	If selected 'yes', APA-Scan will report all the candidate cleavage sites of a gene, whether they are significant or not. Otherwise, APA-Scan will report the most significant event for each gene [default]. Value: yes or no
annotation* :	RefSeq annotation file, downloaded from UCSC Genome Browser, in .txt format
genome* :	Genome fasta file, in .fa format
output_dir :	Output directory for writing the results. [optional]

An example of the congiration.ini file is provided below:

```
[INPUT_RNAseq]
# Input folder names
# All samples(names like sample1_1.bam, sample1_2.bam....) in group1 must be inside of one folder
# All samples(names like sample1_1.bam, sample2_2.bam....) in group2 must be inside of one folder
input1 = /home/input/Group1
input2 = /home/input/Group2

[INPUT_PASseq]
# All samples(names like sample1_1.bam, sample1_2.bam....) in group1 must be inside of one folder
# All samples(names like sample1_1.bam, sample2_2.bam....) in group2 must be inside of one folder
# Default is NULL
pas1 = NULL
pas2 = NULL

[ANNOTATION]
# Put annotation and genome information
annotation = annotation.txt
genome = genome.fa

[Extended_3UTR]
# Run APA-Scan on 'Extended-3UTR' mode
# Value: yes/no. Default is no
extended = no

[All_events]
All = no

[OUTPUT_FOLDER]
output_dir = /home/output_dirname
```

Once the parameters have been specified in the configuration file, the user will open a terminal and enter the following command to run APA-Scan:
$ python3 APA-Scan.py

APA-Scan.py will generate several intermediary files in the output directory. After computing the significance of the association between the two groups of samples, the final results will be writ-

ten in the file named **Group1_Vs_Group2.csv**. The following image shows some of the generated fields in Group1_Vs_Group2.csv:

Chrom	Gene Name	Strand	Start	End	Position	p-value	Ratio Difference	Absolute ratio difference
chr19	RPL13A	+	49491728	49492307	49491826	1.92E-42	-0.065610766	0.065610766
chr10	VIM	+	17237229	17237597	17237318	4.61E-31	0.028446051	0.028446051
chr17	RPL26	-	8377515	8377692	8377564	2.98E-26	-0.04344075	0.04344075
chr5	STC2	-	173314722	173318249	173317376	2.33E-22	0.112542951	0.112542951
chr17	RPL19	+	39204524	39204730	39204650	1.03E-21	-0.049312569	0.049312569
chr13	CDC16	+	114272183	114272726	114272209	4.05E-17	-0.195062219	0.195062219
chr7	HNRNPA2B1	-	26189935	26192577	26191861	5.11E-16	-0.061955041	0.061955041
chr9	RABL6	+	136833719	136834476	136834327	5.68E-15	-0.30708408	0.30708408
chrX	RPS4X	-	72272602	72272772	72272628	2.08E-14	0.041582181	0.041582181
chr1	STMN1	-	25900115	25901087	25900534	2.9E-14	0.043951567	0.043951567
chr17	RPAIN	+	5432541	5433020	5432863	3.79E-14	0.061525048	0.061525048
chr6	SOD2	-	159679063	159682638	159682113	8.77E-14	-0.076980538	0.076980538
chr11	CCDC84	+	119015537	119015792	119015726	2.19E-12	-0.057536734	0.057536734
chr1	SYNC	-	32679905	32681860	32680315	4.57E-12	0.095614951	0.095614951
chr1	RPS8	+	44778575	44778740	44778687	1.21E-11	0.037494068	0.037494068
chr8	SFRP1	-	41261956	41265489	41265472	5.35E-11	-0.292512141	0.292512141
chr15	TPM1	+	63069869	63071914	63069945	5.54E-11	0.075237838	0.075237838
chr1	MEF2D	-	156463720	156467656	156467650	1.5E-10	-0.282933192	0.282933192
chr5	PHYKPL	-	178208473	178211970	178211572	3.28E-10	0.079757735	0.079757735
chr2	RPL37A	+	216501340	216501465	216501446	3.91E-10	0.019741659	0.019741659
chr12	PRIM1	-	56731579	56731734	56731638	8.48E-10	0.190879674	0.190879674
chr18	RMC1	+	23531624	23531807	23531765	9.45E-10	-0.13019906	0.13019906

5 Run Make-plots.py

Make-plots.py also requires the same configuration file to run. It will use the input and output directories listed in the configuration file and prepare a read coverage plot along with the 3'-UTR annotation based on user defined region.
python3 Make-plots.py
After executing this command above for a few seconds, Make-plots.py will ask the user to insert the region of interest in a specific format:
Chrom:GeneName:RegionStart-RegionEnd

5.1 Make-plots.py parameter descriptions

Chrom	Name of the chromosome
GeneName	Name of the gene
RegionStart	Starting position of the region
Region End	End position of the region

Example: **chr1:Tceb1:16641724-16643478**

Make-Plots.py will generate a visual representation of the results shown for each of the regions entered. The plot will illustrate the most significant transcript cleavage site with a red vertical bar on top of RNA-seq read data (and 3'end-seq if available). If the input parameters have 3'end-seq information along with the RNA-seq, then it will generate plots for both cases (See figure below). It will also show the UTR truncation point (annotated and unannotated) at the bottom panel.

The first two subplots of the figure represent the read coverage of the two biological conditions. The bottom subplot shows the gene annotation and the exon information of that gene.

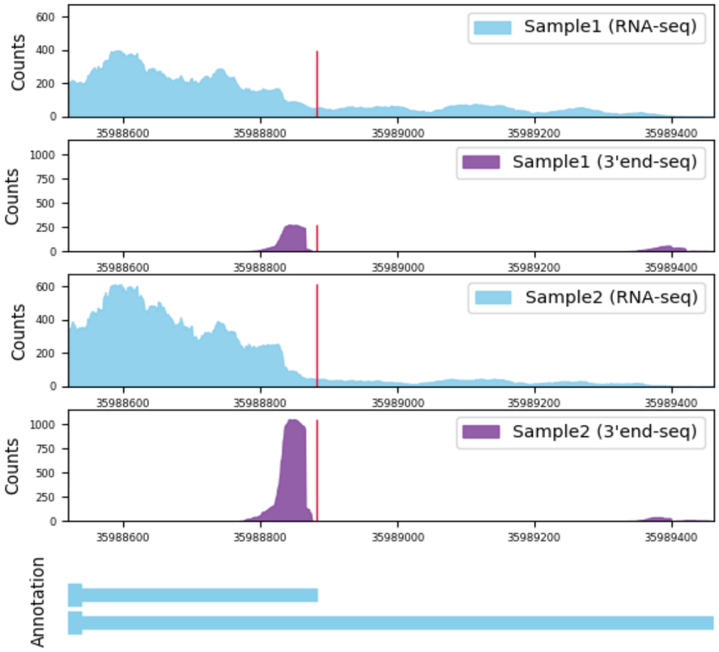
Sample1 (RNA-seq)
Sample1 (3'end-seq)
Sample2 (RNA-seq)
Sample2 (3'end-seq)
Counts
Annotation
35988600
35988800
35989000
35989200
35989400
